# Isolation of entomopathogenic bacteria in the Southwest region of Paraná state in Brazil

**DOI:** 10.1186/1753-6561-8-S4-P253

**Published:** 2014-10-01

**Authors:** Samara Ernandes, Fabiana Maria Castro Da Rosa

**Affiliations:** 1 Universidade Tecnológica Federal do Paraná (UTFPR), Paraná, Brazil

## Background

The use of entomopathogenic bacteria to control harmful insects has increased the interest and researches about spore forming bacteria. Species from genus *Bacillus *are described as rod-shaped cells, sometimes in chains, and mostly capable of producing endospore. Although hundreds of bacteria species are known to be associated with insects, there is only a few which can be used as biological control for agricultural pests as well as, for vectors of tropical diseases.

Several agricultural pests seem to be resistant to both chemical pesticides and known toxins produced by entomopathogenic bacteria. Therefore, the goal of this study was to describe the isolation of an entomopathogenic bacteria contributing with researches for new potentially toxic isolates in biological control of insects.

## Methods

Strains of bacteria were isolated from soil samples of eleven locations. These locations were characterized according to the occupation and the use of the land, and allocated as SISLEG map of Universidade Tecnológica Federal do Paraná - UTFPR - Campus Dois Vizinhos. The method of isolation was the recommended by the World Health Organization, as follows, 1g of each soil sample was placed into sterile tubes with 10mL of saline solution and stirred by vortex for 2 minutes, then 1.5mL were transferred to sterile microtubes and heated during 12 minutes at 80ºC and kept 5 minutes on ice. Samples were then diluted in sterile saline (1000-fold), seeded on Petri dishes with nutrient agar medium and incubated at 30ºC for 48 hours. After incubation, colonies of *Bacillus *spp. were selected [1] by initial identification by differential growth in medium NYSM [2] with penicillin (100mg.L^-1^). The isolates from this medium that presented small crystals of circular and oval size when stained with crystal violet observed with optical microscope (40x) were identified as *Bacillus thuringiensis *[3,4].

## Results and conclusions

Bacteria growth was observed in all eleven soil samples processed in nutrient agar. Heat shock was applied in order to obtain sporulating strains. Colonies were selected according to morphological characteristics standards. Area (1) - *Pinus*, presented more variability with fourteen morphological types (14), followed by area (2) - *Eucalyptus *with nine morphological types. Area (9) - *Pastures *presented the lowest diversity with three types. When a selective entomopathogenic medium was used (penicillin), the area (1) showed extensive growth of strains resistant to this antibiotic, featuring the *Bacillus thuringiensis*, however area (2) showed little growth of colonies.
